# Three-Dimensional Printing of Shape Memory Liquid Crystalline Thermoplastic Elastomeric Composites Using Fused Filament Fabrication

**DOI:** 10.3390/polym15193961

**Published:** 2023-09-30

**Authors:** Peerawat Prathumrat, Mostafa Nikzad, Fareed Tamaddoni Jahromi, Elnaz Hajizadeh, Igor Sbarski

**Affiliations:** 1 Department of Mechanical and Product Design Engineering, School of Engineering, Swinburne University of Technology, Hawthorn, VIC 3122, Australia; ftamaddonijahromi@swin.edu.au (F.T.J.); isbarski@swin.edu.au (I.S.); 2 Department of Mechanical Engineering, Faculty of Engineering and Information Technology, University of Melbourne, Parkville, VIC 3010, Australia; ellie.hajizadeh@unimelb.edu.au

**Keywords:** 3D printing, liquid crystalline thermoplastic elastomer (LCTPE), lignin, composites, fused filament fabrication (FFF)

## Abstract

Liquid crystalline elastomers (LCEs) are stimuli-responsive materials utilised in shape memory applications. The processability of these materials via advanced manufacturing is being paid increasing attention to advance their volume production on an industrial scale. Fused filament fabrication (FFF) is an extrusion-based additive manufacturing (AM) technique that offers the potential to address this. The critical challenge, however, is the rheological characteristics of LCEs that need to be tuned to achieve a facile processability through the extrusion-based method. In this work, new filaments of liquid crystalline thermoplastic elastomer (LCTPE) and its composites with lignin were made by the ternary system of LCE, thermoplastic polyurethane (TPU), and lignin. The results showed that TPU improves the melt flow index of the LCTPE system to approximately 10.01 g/10 min, while adding lignin further enhances the value of this index for the composites up to 21.82 g/10 min. The microstructural analysis indicated that the effective distribution of lignin and reduced domain size of the LCEs in the ternary blend contribute to the enhanced flowability of this filament through 3D printing. Samples of 3D-printed LCTPE and LCTPE/lignin composites maintained their shape memory characteristics via thermomechanical activation. Full shape recovery of the new LCTPE matrix and its composites with lignin was achieved in 39 s and 32 s at 130 °C, followed by 28 s and 24 s at 160 °C, respectively. The successful fabrication of LCTPE and LCTPE/lignin composite samples through 3D printing demonstrates a potential procedure for processing these shape memory materials using the FFF technique, and lignin offers a sustainable and cost-effective material solution that enhances the properties of this composite material.

## 1. Introduction

The additive manufacturing (AM) technique was first developed in 1986 to fabricate a variety of materials with complex geometries from 3D model data [1]. This technology possesses advantages over conventional moulding techniques, such as lead time reduction, low cost, freedom of design, and geometry customisation [2]. Currently, various industries, such as biomedical, prototyping, construction, and polymer composites, exploit this technology to create printed products [3,4,5]. In polymer science and engineering, 3D printing plays a significant role in fabricating polymer parts, in which these materials can be built by different printing techniques, depending on the nature of the crystalline or amorphous structures. Fused filament fabrication (FFF) is one of the most widespread and popular 3D printing techniques to create polymeric samples, which is built on the melt extrusion method. This technique operates by extrusion of the thermoplastic filament into the desired shape according to the defined CAD models [6].

Liquid crystalline elastomers (LCEs) are a particular class of polymeric materials that possess elastic and anisotropic properties [7]. These elastomers can respond to external stimuli such as heat and UV, which can deform their shapes. This stimuli-responsiveness makes them suitable to utilise in shape memory and shape-changing applications. Furthermore, their molecular structures contain mesogens and flexible tails resembling the hard and soft phases of the typical underlying mechanism of the shape memory polymers (SMPs). In our previous work, LCE based on diacrylate reactive mesogens and their relevant composites with lignin were fabricated and exhibited excellent shape memory properties [8]. These elastomeric composites with lignin exhibited shape fixity and shape recovery of 97.0% and 99.7%, respectively. Furthermore, the presence of lignin content in this composite system could reduce the recovery time to 70 s, while the neat LCE sample took 100 s.

In order to exploit these materials on an industrial scale for mass customisation, the processability of these composite materials through 3D printing technology needs to be investigated. In the last decade, several 3D printing techniques based on vat photopolymerization, such as stereolithography (SLA) and direct ink writing (DIW), have successfully fabricated LCE parts, though often at small scales [9]. However, the 3D printing techniques based on fused filament fabrication (FFF) have been given limited attention due to issues associated with the rheological behaviour of LCEs, especially at the macroscopic scale. To obtain suitable rheological properties of LCEs for extrusion-based 3D printing, fabricating hybrid materials containing hard thermoplastic and soft elastomeric phases is a potential solution that provides an alternative approach to printability [10]. Such thermoplastic elastomers (TPEs) give rise to microstructures with tuneable flow properties like thermoplastics, which allows leveraging a straightforward and faster extrusion process based on the well-developed rheological principles for this class of material.

Thermoplastic polyurethane (TPU) is a widely used candidate as a hard-thermoplastic phase in TPE structures. This material is a linear block copolymer with thermoplasticity, elasticity, shear thinning behaviour, 3D printability, and low cost, making it apposite for extrusion techniques [10]. Furthermore, TPU can exhibit shape memory characteristics because of their hard diisocyanate soft polyol segments. Recently, Wang et al. [11] fabricated shape memory polycaprolactone (PCL)/TPU composites using a FFF technique. These materials showed excellent shape fixity and recovery of 100%. Bi et al. [12] utilised a FFF to build shape memory polyurethane/polycaprolactone blends with dopamine/multi-walled carbon nanotubes (PMWCNTs) filler. These materials responded to near-infrared light and exhibited shape fixity and recovery of 90% and 75% after three cycles. In addition, Huang et al. [13] reported that the addition of TPU enhanced the melt flow index (MFI) of the polymer blends due to its high melt fluidity, resulting in an increased flowability for the processing techniques. Given TPU’s suitable molecular mechanism for shape memory applications, these previous works suggest that TPU is an excellent material for improving the rheological properties of polymeric materials for 3D printing through a FFF technique.

Lignin is a bio-based material produced as a by-product of the pulping process. This material has been used as a filler in polymeric composite systems due to its beneficial characteristics—for instance, high thermomechanical properties, high UV stability, renewability, being environmentally benign, and low cost [14]. Recently, Zhou et al. [15] studied the 3D printing of lignin/TPU and carbon fibre (CF)/lignin/TPU composites using a FFF technique. The lignin/TPU composites could be printed and demonstrated a smooth surface up to 50 wt% lignin. The CF was further added at 0.5 wt% to enhance the mechanical properties of the printed composites. Their results showed that the tensile strength and elongation at break of the CF/lignin/TPU composites increased by 67% and 138% compared to lignin/TPU composites.

Considering the superior shape memory characteristics of LCE and lignin/LCE composites, they are great candidates in a variety of industrial and engineering applications, such as in soft robotics, aerospace engineering, smart actuators, and sensors. In order to enable their high-volume production, the processibility of these materials needs to be investigated. Fused filament fabrication (FFF) 3D printing technology has been selected due to being relatively fast, reliable, and inexpensive. However, the rheological properties of LCE are the major challenge that needs to be improved for FFF 3D printing. Thus, this work focuses on investigating the processability of a new liquid crystalline thermoplastic elastomer (LCTPE) filament based on LCE and TPU and their composites containing lignin through the FFF AM technique. The LCE was synthesised using RM257 monomers and PETMP crosslinking agent through a thiol-Michael addition reaction [8]. The TPU was used to improve the printability of the system and contribute to the shape memory behaviour of the printed LCTPE samples. Lignin was further added to create the LCTPE composites. The shape memory behaviour and thermal, rheological, microscopic, and spectroscopic properties of these materials were characterised.

## 2. Materials and Methods

### 2.1. Materials

Diacrylate mesogen, named 1,4-bis4-(3-acryloyloxypropypropyloxy) benzoyl-oxy]-2-methylbenzene (RM257), was purchased from Daken Chemical (Zhengzhou, China). Kraft lignin (alkali lignin), pentaerythritol tetra (3-mercaptopropionate) (PETMP) crosslinking agent, and dipropyl amine (DPA) catalyst were purchased from Sigma-Aldrich, Inc. (Castle Hill, NSW, Australia). Thermoplastic polyurethane (WHT-1180EC) was purchased from Wanhua Chemical Group Co., Ltd. (Yantai, China). Toluene (AR grade) was purchased from ChemSupply Australia Pty Ltd. (Gillman, SA, Australia). All materials were used as received without further purification.

### 2.2. Sample Preparation

Liquid crystalline elastomer (LCE) was synthesised following the steps from our previous work [8]. First, RM257 monomers were dissolved by adding 40 wt% toluene. The mixtures were heated at 80 °C in the oven until becoming a homogeneous solution. The solution was then left to cool down at room temperature. Next, 36 wt% PETMP crosslinking agent and 14 wt% DPA catalyst were added dropwise into the solution. In this work, the DPA was separately diluted in toluene at a weight ratio of 1:50 before adding to the solution. The solution was immediately stirred and vacuumed to remove the bubbles that occurred during the mixing process. The solution was then poured into the mould and left to cure at room temperature for 24 h. Upon completion of the polymerisation process, the LCE samples were heated at 80 °C for 12 h to evaporate the solvent before characterisation.

Liquid crystalline thermoplastic elastomers (LCTPEs) were prepared by mixing LCE with thermoplastic polyurethane (TPU) at a weight ratio of 50:50. TPU was dried at 80 °C for 24 h in a vacuum oven before mixing. These components were mixed using an internal batch mixer (HAAKE Polylab QC, Thermo Scientific, Waltham, MA, USA) at 190 °C with separately different rotor speeds of 60 rpm, 120 rpm, and 180 rpm. Meanwhile, the LCTPE/lignin composites with an LCE:TPU:lignin mass content of 40:40:20 were prepared using a batch mixer in two steps. The details of the mixing process are included in Table 1.

**Figure 1:**
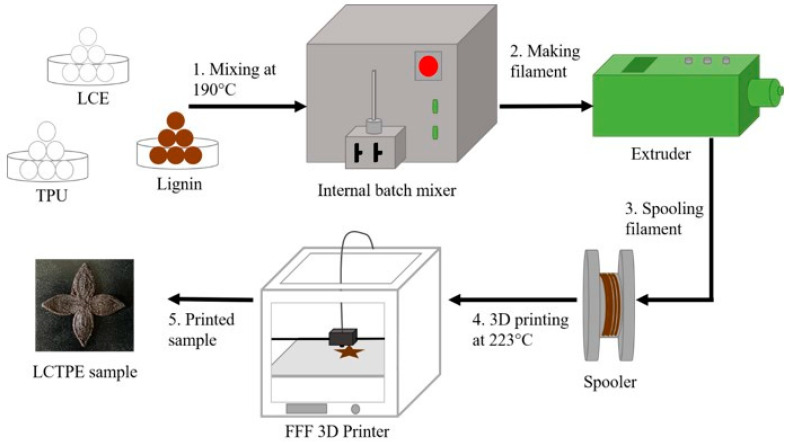
Filament preparation and 3D printing process of LCTPE and LCTPE/lignin composite materials.

The filaments of the LCTPE and LCTPE/lignin composites were prepared using a single-screw extruder, Filabot EX2 (Filabot HQ, Barre, VT, USA). The LCTPE was extruded at 190 °C with an average diameter of 2.85 mm to be used on an Ultimaker^3^ FFF 3D printer. The extruded filament was cooled down using two fans at room temperature and spooled using a Filabot spooler. The processes of filament preparation and 3D printing are shown in Figure 1.

### 2.3. Sample Characterisation

#### 2.3.1. Melt Flow Index

The melt flow index (MFI) of the neat TPU, LCTPE, and LCTPE/lignin composites was measured using the melt flow index tester Modular Line (CEAST, Instron, Pianezza, Italy). A sample mass of 8 mg to 10 mg was used. The test was performed at 200 °C under a specific load of 2.16 kg.

#### 2.3.2. Optical Microscopy

The surface morphology and particle distribution of the LCTPE and LCTPE/lignin composite samples were investigated using optical microscopy (Olympus BX61, Hamburg, Germany). The images of these samples were produced by imaging at 20× magnification. A 50 μm × 50 μm ROI was set for the best focus of the area of each captured image.

#### 2.3.3. Thermal Properties

Differential scanning calorimetry (DSC) measurements were conducted to evaluate phase transition temperatures of the neat TPU, neat LCE, LCTPE samples, and relevant composites using a DSC 2960 (TA Instruments, New Castle, DE, USA). The samples with a mass of 5 mg to 10 mg were sealed in aluminium pans. The heating rate was set at 5 °C/min in a temperature range of −40 °C to 150 °C. A N_2_ atmosphere was set with a 110 mL/min flow rate.

#### 2.3.4. Thermal Stability

The thermal stability of all the materials was evaluated using a thermogravimetric analyser (TGA) Q50 (TA Instruments, New Castle, DE, USA). A sample weight of 10 mg to 20 mg was used. All samples were run with a heating rate of 10 °C/min from room temperature to 800 °C. The air was purged with a flow rate of 60 mL/min. Thermal degradation and derivative thermogravimetric (DTG) curves of all the samples were analysed.

#### 2.3.5. Fourier-Transform Infrared (FTIR) Spectroscopy

FTIR analysis was used to measure the structural composition of neat TPU, neat LCE, LCTPE, and their composites with lignin. The FTIR Nicolet iS5 (Thermo Scientific) was utilised with a scan range of 4000 cm^−1^ to 400 cm^−1^. The spectra were scanned in a transmittance mode with 64 scans per sample.

#### 2.3.6. Shape Memory Behaviours

Shape memory behaviours of the LCTPE and composite samples were observed, including shape fixity and shape recovery. The samples were heated at 130 °C for 10 min and manually deformed to a temporary shape. Then, the samples were cooled down to room temperature to maintain this temporary shape. The samples were reheated at 130 °C to observe their shape recovery. The images of various stages of these samples were taken through a shape memory process. In addition, the effect of temperature on the recovery time of these samples was recorded using a video camera at temperatures of 130 °C and 160 °C.

## 3. Fabrication of 3D-Printed LCTPEs and Their Composites

**Figure 2:**
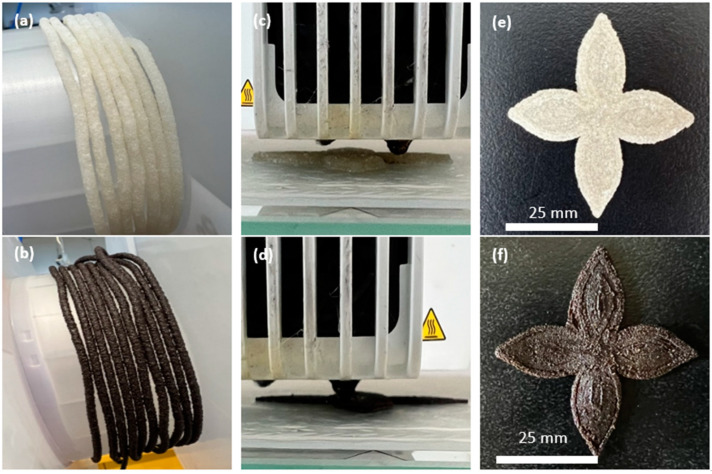
The filaments of the LCTPE@180 and LCTPE/lignin@180 composites (**a**,**b**), FFF 3D printing process (**c**,**d**), and printed specimens with a thickness of 2 mm (**e**,**f**).

The flower-shaped samples of the LCTPE@180 and LCTPE/lignin@180 composites were printed using a FFF machine, Ultimaker^3^ (Zaltbommel, The Netherlands). Slicer software Cura 4.13 was used to prepare the G-code file. The process parameters for 3D printing the samples are summarised in Table 2. All samples were printed with 100% infill density using concentric patterns. A nozzle temperature of 223 °C was set to enhance the extrusion ability and avoid clogging [16]. The viscosity of the polymer composite melt was shown to be proportional to the reciprocal of the absolute temperature, which was governed by the activation energy of the system denoted by Arrhenius’s Equation [17]. The filaments, printing process, and examples of printed samples are shown in Figure 2.

## 4. Results and Discussion

### 4.1. The Effect of Rotor Speed on the Melt Flow Index of LCTPE and Their Composites

The melt flow index (MFI) is a measure of the flow characteristics of the molten polymer under a particular shear stress. This property is a crucial parameter in providing insight into the printability of the polymeric samples using the extrusion-based printer. In a previous study, the MFI threshold value of 10 g/10 min was sufficient for printing polylactic acid (PLA) through a FFF technique at a temperature range of 190 °C to 220 °C [18]. In this work, the MFI of neat TPU and LCTPE and composite samples were evaluated at 200 °C using an applied load of 2.16 kg.

**Figure 3:**
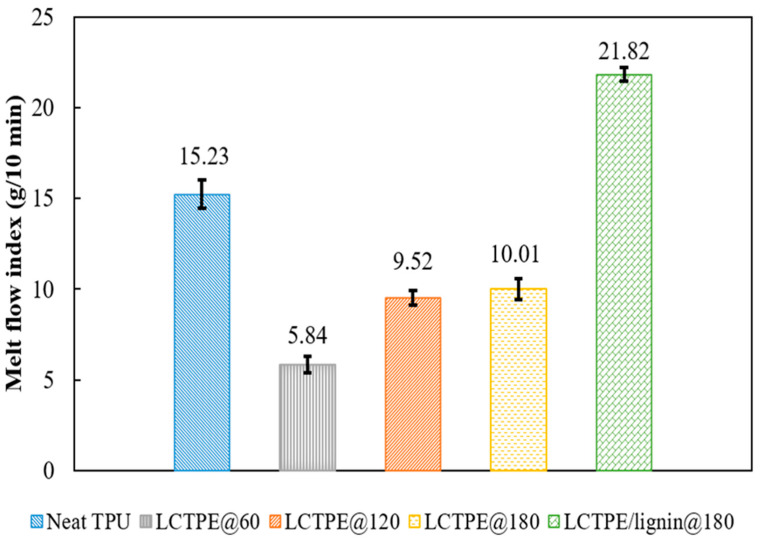
Melt flow index (MFI) test results of the neat TPU, LCTPE@60, LCTPE@120, LCTPE@180, and LCTPE/lignin@180 composite samples at 200 °C.

Figure 3 demonstrates that the MFI of the LCTPEs increases with the increasing rotor speed in the mixing process. In this study, the LCTPE samples were mixed with different rotor speeds of 60 rpm, 120 rpm, and 180 rpm. The LCTPE samples mixed at 60 rpm (LCTPE@60), 120 rpm (LCTPE@120), and 180 rpm (LCTPE@180) displayed MFI values of 5.84 ± 0.46 g/10 min, 9.52 ± 0.42 g/10 min, and 10.01 ± 0.58 g/10 min, respectively. This increment could be attributed to a decreased particle size of the LCEs at a higher shear rate, which results in an increased MFI value of the LCTPE material at a high temperature. An increase in the MFI value of the LCTPE samples also contributed to their printability through the extruder nozzle of the printer. These results were similar to those reported by Tjong et al. [19]. In addition, the LCTPE@180 sample showed a continuous filament and smooth surface after the filament extrusion process. Meanwhile, the MFI value of neat TPU was 15.23 ± 0.79 g/10 min. This value was higher than the LCTPE samples, in which the strong covalent bonding of the crosslinked LCE structures in the LCTPE matrix reduced their flowability at elevated temperatures. In contrast, the MFI value of the neat LCE material could not be measured, since it could not be melted at the set temperature of the MFI test due to the high stiffness and strength of its crosslinked structures.

Furthermore, the LCTPE/lignin composites were prepared with a mass ratio of 40:40:20 in a batch mixer with a rotor speed of 180 rpm. The incorporation of lignin into the LCTPE matrix increased their MFI value. The results showed the MFI of LCTPE/lignin@180 composites of 21.82 ± 0.37 g/10 min. It could be suggested that lignin served as a plasticiser at elevated temperatures, increasing the flowability of the composite materials [20].

### 4.2. Surface Morphology of LCTPE and Composite Materials

**Figure 4:**
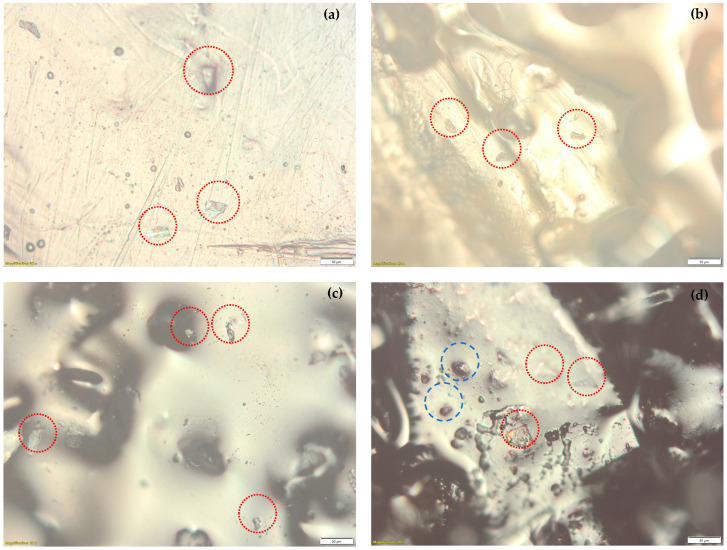
Optical microscopy images of (**a**) the LCTPE@60, (**b**) LCTPE@120, (**c**) LCTPE@180, and (**d**) LCTPE/lignin@180 composites magnified 20×; LCE particles (red dotted circle) and lignin (blue dashed circle).

The surface morphology of the LCTPE samples fabricated by different rotor speeds and the LCTPE/lignin composite sample was investigated using an optical microscope. The microscope images of various samples are shown in Figure 4a–d. The image of the LCTPE@60 sample in Figure 4a shows the crystalline morphology of the LCE particles distributed along the TPU region. An increase in rotor speed in the mixing process reduced the particle size of the LCE, as seen in Figure 4b,c. A high centrifugal force of a high rotor speed in the mixing process could improve the shear behaviour of the LCE domain, resulting in reduced particle sizes. It could confirm an increase in the MFI values of these materials, where the reduced LCE particles could enhance the flowability of the molten LCTPE through the nozzle without clogging. The addition of a lignin filler to the LCTPE matrix in Figure 4d showed the agglomeration characteristic due to the strong cohesion force between the lignin particles [21] and moderate dispersion. Nevertheless, lignin tended to distribute along the LCTPE matrix. This filler significantly improved the MFI value of the LCTPE/lignin@180 composite material, further contributing to the flowability of these composites. Thus, the size reduction of the LCE particles and distribution of lignin along the LCTPE matrix could improve the flow behaviour, leading to 3D printability via a FFF technique.

In addition, the figures indicate the immiscible blending of various compositions. To consider the dynamics of the heterogeneous blending of this composite system, the size and size distribution of the dispersed phase in the polymer matrix play an essential role [22]. The average crystalline morphology size of LCE is likely to decrease with a higher shear rate, which enhances the rheological properties of the composites. The topology and convection flow of the configuration of the interfaces are other influential factors in the dynamics of the blending [23]. The aromatic structure of the lignin filler also enables an increase in the flowability of the blend when replacing the linear structures of the LCE/TPU phase due to the improved imposed flow field. This is similar to the report of Chapman et al. [24]. Meanwhile, the modification of the interface of crystalline blending with lignin as a complex structural filler results in its viscoelastic response, in which the interfacial region is enabled to slip by the applied shear stress, similarly reported by Galeski [25] and Saha et al. [26]. In addition, the interfacial dynamics of the polymers can be varied by a degree of stretching and molecular weight [27].

### 4.3. Thermal Properties of LCTPEs and Their Composites

**Figure 5:**
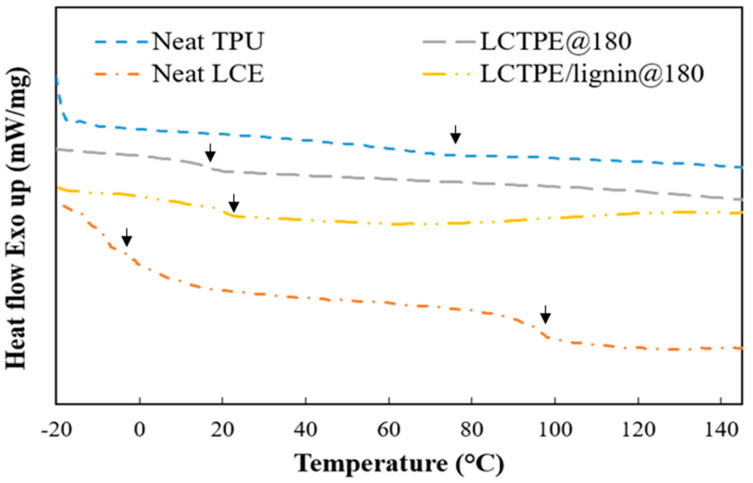
DSC thermograms of neat LCE, neat TPU, and LCTPE materials in oxidative conditions.

The thermal properties of the neat LCE, neat TPU, LCTPE@180, and LCTPE/lignin@180 composites were evaluated using DSC analysis. Figure 5 illustrates the thermogram of these samples over well-defined temperatures. The neat LCE exhibited two phase transitions at −6 °C and 96 °C, corresponding to a glass transition (T_g_) and a nematic–isotropic transition (T_NI_) temperature. The T_NI_ is a particular transition temperature of the LCE class of polymers that allows the molecular alignment of liquid crystalline structures from nematic to isotropic states. This temperature can be used as a switching temperature in a shape memory process without interference by ambient conditions. The DSC curve of the neat TPU exhibited a small endothermic peak at approximately 70 °C. This peak can be defined as the melting temperature of hard-segment domains of diisocyanate structures [28].

In addition, a DSC curve of the LCTPE@180 sample was evaluated. This material indicated a T_g_ at 16 °C, which was higher than the neat LCE sample. This enhancement could be attributed to the effect created by the rigid diisocyanate segments of urethane structures. However, the absence of an endothermic peak of the neat TPU in the LCTPE@180 curve was possibly due to the lack of intermolecular interactions between hard segments of TPU, since this segment could form a hydrogen bond with lignin [29]. Furthermore, the LCTPE/lignin@180 composites exhibited two inflexion points of stepwise transition at 25 °C and 95 °C, corresponding to a T_g_ and T_NI_, respectively. The T_g_ of the LCTPE/lignin@180 composites was slightly shifted higher than the LCTPE@180 sample. This could be explained by the fact that the three-dimensional network structures of the lignin content could obstruct the movement of the polymeric chains of the LCTPE matrix, resulting in an increased T_g_.

### 4.4. Thermal Stability of LCTPEs and Their Composites

**Figure 6:**
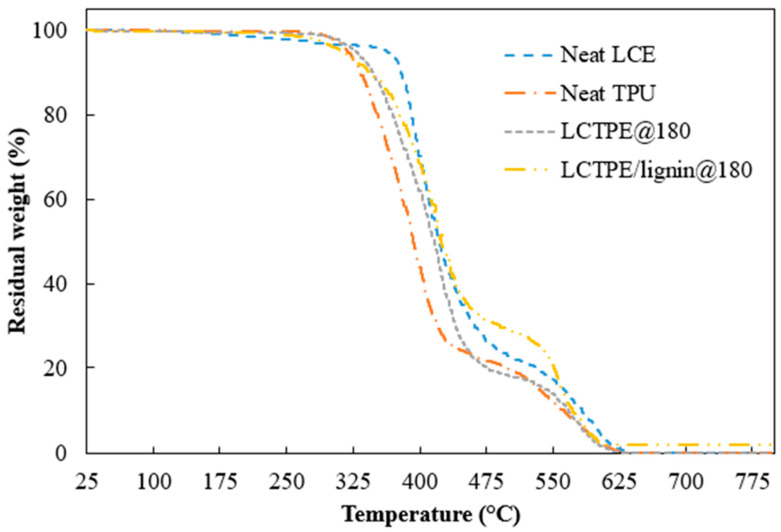
Thermal degradation of neat LCE, neat TPU, and LCTPE materials in oxidative conditions.

**Figure 7:**
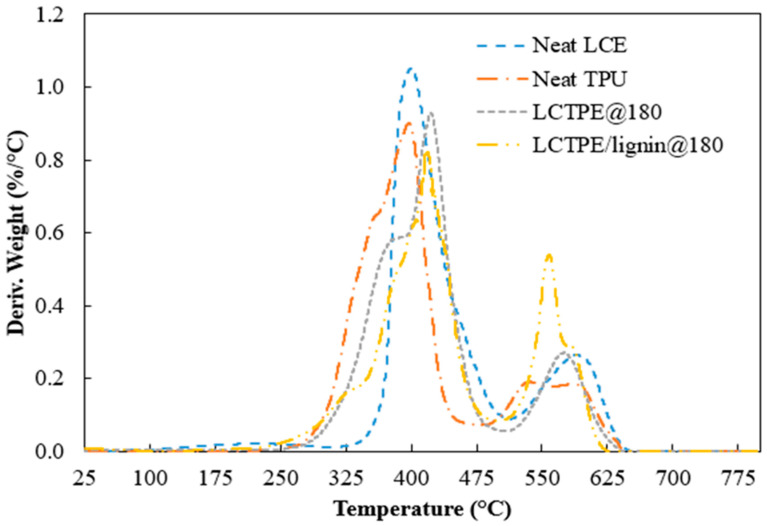
DTG thermograms of neat LCE, neat TPU, LCTPE@180, and LCTPE/lignin@180 composite materials in oxidative conditions.

The thermal stability of the neat LCE, neat TPU, LCTPE@180, and LCTPE/lignin@180 composite samples were examined under oxidative conditions. The TGA and DTG thermograms of all the samples are depicted in Figure 6 and Figure 7. All the samples exhibited two degradation stages. The neat LCE sample showed the degradation temperature at 10 wt% loss (T_d,10_) at 379 °C and the first maximum DTG peak at 403 °C. This degradation stage was ascribed to the breakages of the thioether linkages of the LCE structures and the decomposition of the aliphatic chains of RM257. The second DTG peak of LCE was indicated at 590 °C, corresponding to the fragmentation of the nematic mesogens of the LCE structures. Meanwhile, the TGA curve of the neat TPU showed the T_d,10_ at 333 °C and the first DTG peak at 400 °C, which corresponded to the scission of the hard segment with the formation of diisocyanate and diol groups. The second DTG peak was in the range of 530 °C to 598 °C, corresponding to the degradation of the soft diol segment of TPU [30,31].

The LCTPE@180 sample demonstrated a similar pattern of degradation of LCE and TPU. However, the first prominent peak of this material exhibited a synergistic characteristic compared to their parental materials, which could be due to the topological entanglement of the LCE network structures and aliphatic backbones of TPU. The LCTPE/lignin@180 composites exhibited a similar trend as the LCTPE@180 sample. However, the second DTG peak of the composites was higher than the neat LCE, the neat TPU, and LCTPE@180 samples, which could be due to the fragmentation of the phenylpropanoid structures of lignin.

### 4.5. FTIR Analysis of the Neat LCE, Neat TPU, and LCTPE Materials

**Figure 8:**
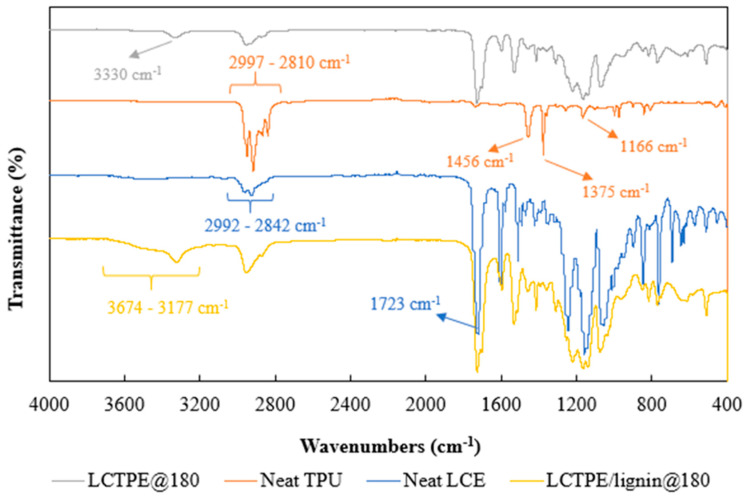
FTIR spectra of the neat LCE, neat TPU, and LCTPE materials.

The FTIR spectra of the neat LCE, neat TPU, LCTPE@180, and LCTPE/lignin@180 composite samples are shown in Figure 8. The spectrum of neat TPU showed peaks in the range of 2997 cm^−1^ to 2810 cm^−1^, indicating asymmetric and symmetric C-H bond stretching vibrations [32]. The peaks at 1456 cm^−1^ and 1375 cm^−1^ belonged to the C-C stretching of aromatic rings and the C-N bond stretching, respectively. The peak observed at 1166 cm^−1^ corresponded to the NH–(C=O)–O bond stretching vibration of urethane groups [33].

The FTIR curve of neat LCE exhibited a broad peak in a range of 2992 cm^−1^ to 2842 cm^−1^, corresponding to intramolecular C-H bond stretching. The peak at 1723 cm^−1^ indicated a bond stretching of C=O in RM257 structures. The peaks between 1300 cm^−1^ and 1000 cm^−1^ specified the C-O-C stretching vibration of the ester groups in the LCE structures. Meanwhile, the LCTPE@180 sample exhibited combined FTIR peaks of LCE and TPU. That combination has no further significant peaks that have been observed. It suggests that the mixture of LCE and TPU was only a result of the physical entanglement between their respective macromolecules. Such an inter-dispersion, however, contributed to a higher shift of the DTG peak of LCTPE@180, which was higher than the neat LCE and TPU, as seen in Section 4.4. A small broad peak at 3330 cm^−1^ of the LCTPE@180 spectrum was further observed, demonstrating a stretching vibration of the N-H groups of the urethane linkages. The spectrum of LCTPE/lignin@180 composites showed a similar curve to the LCTPE@180 sample. However, a broad peak observed between 3674 cm^−1^ and 3177 cm^−1^ corresponded to the O−H stretching of the hydrogen-bonded aromatic and aliphatic groups of lignin structures.

### 4.6. Shape Memory Behaviours of LCTPE Materials

The shape memory behaviours of the 3D-printed LCTPE sample and LCTPE/lignin@180 composites with a thickness of 2 mm were observed through a thermomechanical process, including shape fixity and shape recovery. The molecular structure of LCTPE could underpin the typical molecular mechanism of shape memory polymers (SMPs), in which they consist of netpoints (hard phase) and switch units (soft phase). In the LCTPE system, the mesogen of the LCE structure, together with the diisocyanate segment of TPU, work as netpoints, which are responsible for determining the original shape of the shape memory elastomers. These netpoints can store the elastic energy during the shape memory process. In contrast, the flexible aliphatic chains of the LCE structure and polyol segments of TPU work as switch units in the mechanism, which are responsible for deforming and recovering shapes. Therefore, the combined structures of LCE and TPU contribute to the underlying shape memory mechanism of the LCTPE system. A similar observation was reported by Ghosh et al. [34] that the interpenetrated network of polystyrene/thermoplastic polyurethane-based nanocomposites could decrease the recovery time. This composite system exhibited a full recovery in 22 s and 98 s when activated by a temperature of 50 °C and sunlight at 42 °C, respectively. In addition, the molecular entanglement between LCE and TPU structures could improve the shape recovery of this polymeric system, in which the entanglement could serve as the physical crosslinks and increase the orientational entropy of this system, resulting in the fast and facile recovery over the transition temperature. Similar observations were also made by Hao et al. [35] and Hoy et al. [36].

**Figure 9:**
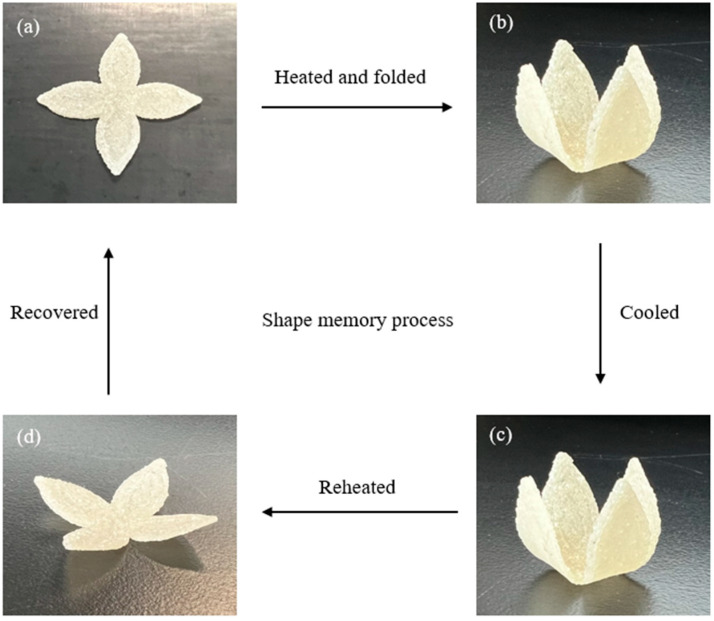
Snapshots of the shape memory process of the LCTPE@180 sample: (**a**) the original blooming shape, (**b**) the temporary shape folded at 130 °C, (**c**) the temporary shape cooled at room temperature, and (**d**) the recovered shape at 130 °C.

**Figure 10:**
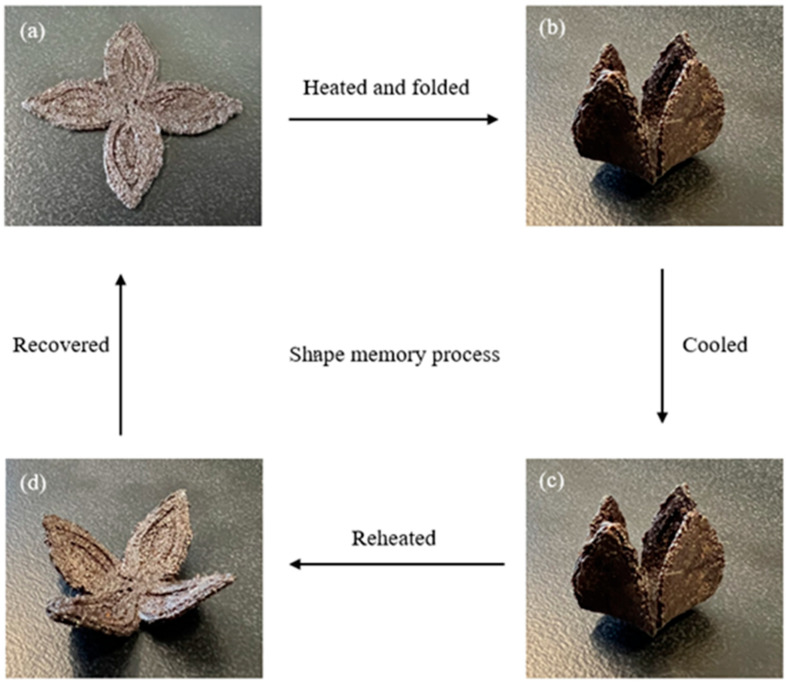
Snapshots of the shape memory process of LCTPE/lignin@180 composite sample: (**a**) the original blooming shape, (**b**) the temporary shape folded at 130 °C, (**c**) the temporary shape cooled at room temperature, and (**d**) the recovered shape at 130 °C.

Figure 9 and Figure 10 illustrate the snapshots of various stages of shape memory behaviours of the 3D-printed LCTPE@180 and LCTPE/lignin@180 composite samples. First, the original blooming shape was heated in the oven at 130 °C for 10 min, where this temperature was above the T_NI_ of this LCTPE system. Second, the sample was then manually folded and cooled to room temperature to maintain the temporary shape. Third, the temporary folded shape was reheated at the same temperature without the applied force. Fourth, the folded sample gradually recovered to a bloomed shape again.

**Figure 11:**
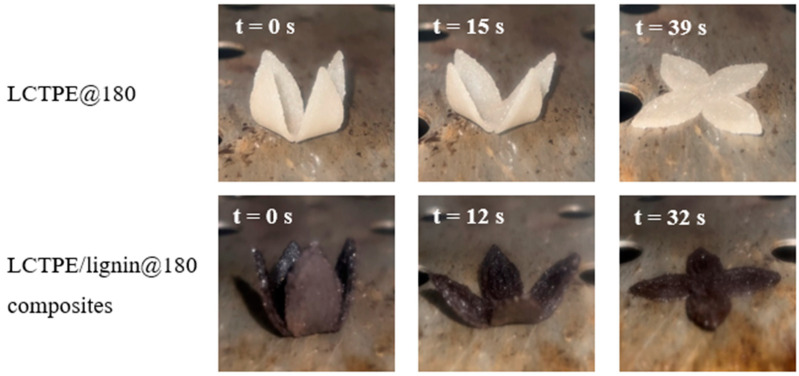
The snapshots of the shape recovery behaviour of LCTPE@180 and LCTPE/lignin@180 composite samples at a temperature of 130 °C.

**Figure 12:**
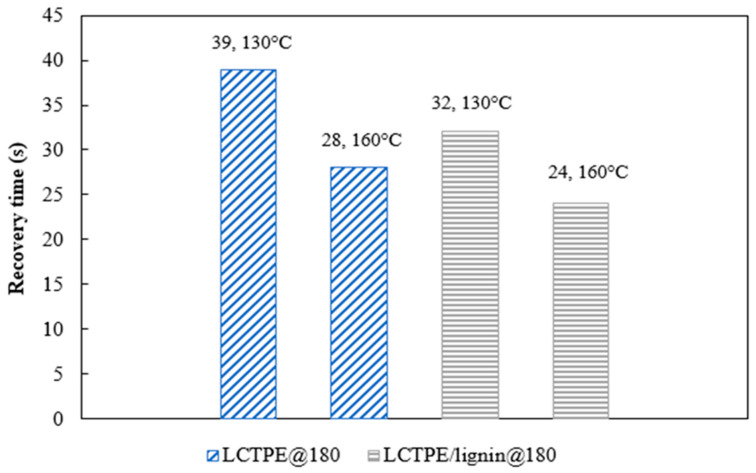
The recovery time of LCTPE@180 and LCTPE/lignin@180 composite samples at different testing temperatures of 130 °C and 160 °C.

The recovery time of these samples was also investigated by the influence of well-defined temperatures using a video camera. Temporarily folded samples were placed in the oven at different temperatures of 130 °C and 160 °C. The snapshots of the shape recovery behaviour at a temperature of 130 °C are shown in Figure 11. Moreover, the results of the recovery time as a function of temperature are plotted in Figure 12. The LCTPE@180 sample exhibited a full recovery in 39 s and 28 s when activated at 130 °C and 160 °C, respectively. However, the LCTPE/lignin@180 composites could fully recover at 130 °C and 160 °C in 32 s and 24 s, respectively. These results indicate that lignin served as the extra netpoint density, which could store the higher elastic energy in the deformation process and release this energy during the recovery process, resulting in the faster recovery of the composites.

## 5. Conclusions

This study succeeded in the 3D printing of a new liquid crystalline thermoplastic elastomer (LCTPE) and their composites with lignin using a FFF technique. The MFI results of LCTPE filaments showed an increasing trend with the increasing rotor speeds of the mixer. The LCTPE@180 filament exhibited a MFI value above 10 g/10 min, corresponding to the threshold value for printing with a FFF printer. Moreover, the addition of a lignin filler into this material further increased the MFI value, confirming the printability of this composite material by using this printing technique. The optical microscopic results demonstrated that an increase in the rotor speed reduced the particle size of LCE crystalline morphology and improved the distribution of lignin along the LCTPE matrix, ensuring the flowability of molten LCTPE/lignin composite materials through the nozzle without clogging. The presence of a lignin filler also increased the glass transition temperature of the composites compared to the neat LCTPE counterpart.

The samples of 3D-printed LCTPE@180 and LCTPE/lignin@180 composites demonstrated excellent shape memory behaviour through thermomechanical activation. A variation in temperature also influenced the recovery time of these materials, in which they could take a shorter time to recover when the temperature increased. Hence, incorporating lignin in the fabrication of LCTPE and its composite samples can be an effective way to improve the printability and processability of this class of shape memory materials for industrial and engineering applications, such as soft robotics, smart sensors and actuators, and high-end space engineering. The utilisation of abundant lignin enables the prospect of developing interesting applications of this exciting class of material in a sustainable and cost-effective fashion. To further advance the functionalities of these materials in future works, the variations of the compositions of this ternary system should be considered.

## Figures and Tables

**Table 1:** The details of the mixing process of LCTPE and LCTPE/lignin composite samples.

Samples	Mixing Process
LCTPE@60	60 rpm for 10 min at 190 °C
LCTPE@120	120 rpm for 10 min at 190 °C
LCTPE@180	180 rpm for 10 min at 190 °C
LCTPE/lignin@180	LCE and TPU were mixed at 180 rpm for 9 min, and lignin was then added to mix for 1 min at 190 °C.

**Table 2:** The parameters utilised for 3D printing of LCTPE and its composite samples.

Parameters	Value
Print nozzle diameter (mm)	0.8
Nozzle temperature (°C)	223
Bed temperature (°C)	50
Layer thickness (mm)	0.15
Printing speed (mm/s)	30

## Data Availability

The data presented in this study are available on request from the corresponding authors.
